# The trypan blue cellular debris assay: a novel low-cost method for the rapid quantification of cell death

**DOI:** 10.1016/j.mex.2019.05.010

**Published:** 2019-05-15

**Authors:** Paul F. Lebeau, Jack Chen, Jae Hyun Byun, Khrystyna Platko, Richard C. Austin

**Affiliations:** Department of Medicine, Division of Nephrology, McMaster University, St. Joseph’s Healthcare Hamilton and Hamilton Centre for Kidney Research, Hamilton, Ontario L8N 4A6, Canada

**Keywords:** Trypan blue cellular debris assay, Cell death, Assay, Trypan blue, LDH assay, Cytotoxicity, Dye, Cell viability, 96-well plate, Apoptosis

## Abstract

Cell death is a common driver of human disease and is frequently studied in a variety of *in vitro* settings. There currently exists a range of commercially available assays to examine cell death, however, most are costly and require assay-specific experimental conditions that may not be suitable for many cell types. Here, we show that cellular debris occurring as a result of cell death can be used to quantify cell death using trypan blue. Furthermore, we demonstrate that the data generated using this technique are comparable to the widely-used lactate dehydrogenase (LDH) assay. Overall, we describe a novel application for trypan blue, a stain found in most biology laboratories, as a novel and cost-effective method for the quantification of cell death via staining of cell debris.

## Graphical abstract

**Figure:**
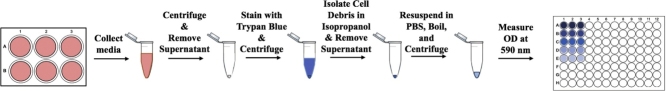

• The technique is quick and affordable.
• No manipulation of experimental conditions, such as low FBS, are required to accommodate the assay.
• Data generated using the assay are consistent with the widely-used LDH release assay.

**Specifications Table**

**Table:** 

Subject Area:	Biochemistry, Genetics and Molecular Biology
More specific subject area:	Molecular Biology
Method name:	Trypan Blue Cellular Debris Assay
Name and reference of original method:	W. Strober, Trypan blue exclusion test of cell viability, Curr Protoc Immunol Appendix 3 (2001) Appendix 3B.
Resource availability:	• 6-well tissue culture dish • 96-well plate • Complete cell culture medium • Phosphate-buffered saline (PBS), pH 7.4 • 99% v/v Isopropanol • 0.4% w/v Trypan blue solution (ThermoFisher Scientific, Cat. no. 15250061) - Alternatively, dissolve 400 μg of trypan blue powder (Sigma- Aldrich, Cat. no. T6146) in 100 mL of distilled water • Microcentrifuge • Dry block heater • 1.5 mL Microcentrifuge tubes • Spectrophotometer

## Method details

Here, we describe a non-invasive method, referred to as the trypan blue cellular debris (TBCD) assay, to examine media content of cell debris occurring as a result of cell death. Following the harvest of debris, live adherent cells can continue to be grown or be utilized for additional assays. Although the following protocol was optimized using 6-well tissue culture dishes, the protocol can also be optimized for use with other culture dish formats.

Equipment and reagents

• 6-well tissue culture dish
• 96-well plate
• Complete cell culture medium
• Phosphate-buffered saline (PBS), pH 7.4
• 99% v/v Isopropanol
• 0.4% w/v Trypan blue solution (ThermoFisher Scientific, Cat. no. 15250061)
  - Alternatively, dissolve 400 μg of trypan blue powder (Sigma-Aldrich, Cat. no. T6146) in 100 mL of distilled water
• Microcentrifuge
• Dry block heater
• 1.5 mL Microcentrifuge tubes
• Spectrophotometer

Seeding and treatment of cells

1 Centrifuge complete medium at 15,000×*g* for 5 min to remove any particulates found in the FBS and warm medium to the desired temperature prior to seeding. This can be done using sterile microcentrifuge tubes in a table-top microcentrifuge.
2 Seed cells in 6-well tissue culture dishes containing the appropriate volume of medium. We recommend using 1 mL of complete medium per well and a seeding density of approximately 8 × 10^5^ cells per well or 80% confluency. Ensure that each well contains an equal number of cells.
3 Treat cells with appropriate drugs/interventions.

Staining of cell debris

**Fig. 1:**
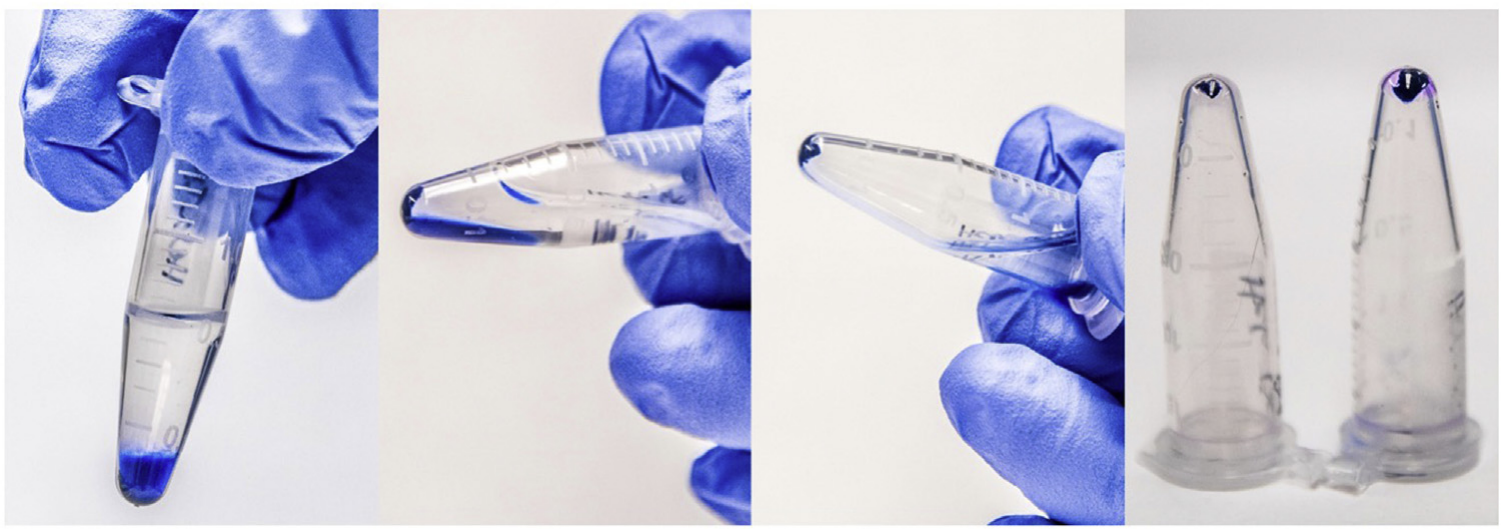
Isolate and wash the trypan blue-stained cell debris pellet by adding 500 μL of isopropanol and gently inverting the tube. The isopropanol will displace the aqueous trypan blue fraction and leave behind a clean blue pellet.

1 Following the desired treatment period, carefully harvest cell debris by collecting all of the medium from each well using a pipette without disturbing cells attached to the bottom of the plate. The medium containing cell debris should be placed in pre-labelled microcentrifuge tubes. Continue to grow the cells by adding fresh pre-warmed medium or collect lysate for analysis of protein or mRNA using the appropriate lysis buffer.
2 Pellet cell debris via centrifugation at 10,000 × *g* for 2 min at room temperature. Although not necessary for most cell types, live weakly adherent cells must be cleared from the media prior to isolation of cell debris via low-speed centrifugation at 500 × *g* for 1 min. Remove the cell debris-containing supernatant and continue the standard protocol; be careful not to agitate the pellet of live cells from the bottom of the tube.
3 Discard the medium by inverting the tube or collect medium for other assays using a pipette. Be careful not to agitate the cell debris pellet.
4 Add 100 μL of trypan blue solution to each tube and re-suspend the pellet using a vortex. Allow cell debris to stain on the bench top for 5 min at room temperature.
5 Centrifuge the samples at 12,000 × *g* for 2 min at room temperature and discard excess trypan blue stain by inverting the tube. Be careful not to disturb the pellet.
6 Wash the pellet by adding 500 μL of 99% isopropanol. Slowly invert the tube and open the lid to remove the wash (Fig. 1). Remove any excess isopropanol using a pipette while being careful not to disturb the trypan blue-stained cell debris pellet.

Extraction and quantification

1 Add 100 μL of PBS to each of the tubes in order to extract the stain from the pellet. Agitate the pellet using a vortex and heat the tubes using a dry block heater for 10 min at 80 °C.
2 Centrifuge the samples to remove cell debris from the blue-coloured PBS extract.
3 Load the blue-coloured PBS extract into a 96-well plate as technical duplicates of 45 μL and measure the optical density of the extract using a spectrophotometer at a wavelength of 590 nm.

### Data analysis and recommended controls

Similar to many assays, data generated using the TBCD assay described in this report can be presented as (a) relative or fold changes in cell death or (b) absolute cell death. To present data in a manner relative to an untreated or vehicle control group, no assay controls are required; simply normalize the mean optical density value obtained from each treatment group to the mean value obtained for the untreated/vehicle control. Error values should be treated in the same manner as the means. To illustrate absolute cell death, blank and 100% cell death controls are required. The blank can be generated by adding 100 μL of trypan blue to a new microcentrifuge tube containing no cell debris (starting at step 4 of Staining of cell debris). A small amount of stain will adhere to the plastic and generate non-specific signal. Optical density generated from the blank should be subtracted from the mean value of each treatment group during data processing. To generate a 100% cell death control, place a culture dish containing the same total number of cells as those used for the treatment groups into a freezer for 20 min; remove medium from these cells prior to freezing. Following freezing, resuspend cell debris in PBS. Ensure that all cells/debris are collected by scraping the bottom of the well with a cell scrapper. Collect and stain these cell debris using the protocol described above. To calculate percent cell death, use the optical density values obtained from experimental and controls groups in Eq. (1).

(1) $$\begin{array}{c} \text{\%}\text{C}\text{e}\text{l}\text{l}\;\text{D}\text{e}\text{a}\text{t}\text{h}=\;\frac{\text{E}\text{x}\text{p}\text{e}\text{r}\text{i}\text{m}\text{e}\text{n}\text{t}\text{a}\text{l}\;\text{V}\text{a}\text{l}\text{u}\text{e}-\;\text{B}\text{l}\text{a}\text{n}\text{k}\;\text{V}\text{a}\text{l}\text{u}\text{e}}{100\text{\%}\;\text{C}\text{e}\text{l}\text{l}\;\text{D}\text{e}\text{a}\text{t}\text{h Value}-\text{B}\text{l}\text{a}\text{n}\text{k}\;\text{V}\text{a}\text{l}\text{u}\text{e}}\;\times 100 \end{array}$$

## Method validation

To ensure the validity and sensitivity of the TBCD assay, HuH7 immortalized human hepatocytes were seeded in 6-well culture dishes at a confluency of 80%. The following day, cell debris was generated by freezing the cells at -80 °C for 20 min. Serial two-fold dilutions of cell debris were carried out prior to staining in order to mimic incremental changes in cell death.

**Fig. 2:**
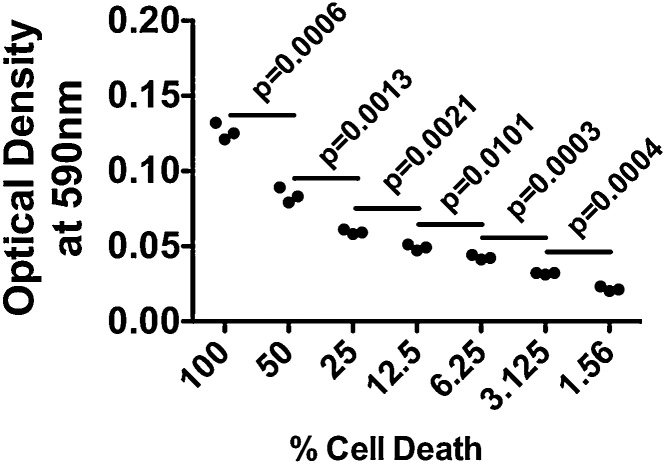
Cell debris was generated by freezing cell monolayers for 20 min (n = 3). Debris was resuspended, underwent two-fold serial dilutions and was stained using the trypan blue debris assay protocol. Statistical analysis was carried out using the unpaired Student’s *t*-test.

**Fig. 3:**
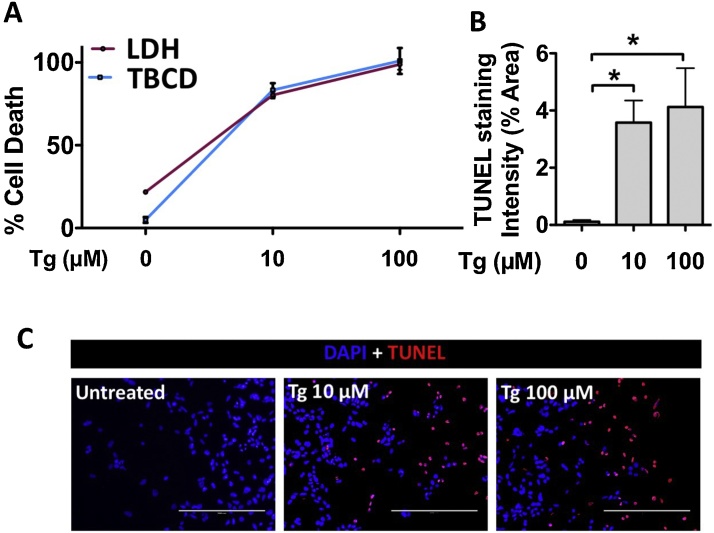
(A) A comparison of data generated using a lactate dehydrogenase (LDH) release assay and the trypan blue cellular debris assay (TBCD) in HuH7 immortalized human hepatocytes treated with thapsigargin (Tg). (B) Apoptotic cells were also visualized by staining for DNA damage using a TUNEL assay, which was quantified using ImageJ Software. Error bars are represented as SD. Scale bars, 200 μm. *, p < 0.05. Statistical analysis was carried out using the unpaired Student’s *t*-test.

Quantification of the optical density of the PBS extracts using a spectrophotometer (Molecular Devices) demonstrates that changes in cell debris concentration can be detected using trypan blue (n = 3 wells) (Fig. 2). HuH7 cells were then treated with increasing concentrations of a pharmacologic agent known to cause cell death in a manner dependent on endoplasmic reticulum stress, thapsigargin (Tg) (Fig. 3A) [1]. Data generated using the TBCD assay were compared to data generated using an LDH release assay (Roche); both assays were carried out on the same experiments using the same medium. Qualitative assessment of apoptosis was also carried out in Tg-treated cells using a terminal deoxynecleotidyl transferase dUTP nick end labeling (TUNEL) assay (Trevigen). TUNEL-positive apoptotic cells were visualized using a fluorescent microscope (EVOS, ThermoFisher Scientific). Quantification of TUNEL staining intensity of 5 separate wells of cells per treatment using ImageJ software (NIH) also reveals that findings are consistent with the LDH and TBCD assays (Fig. 3B and C). Our research group has also used the TBCD assay described in this report to demonstrate changes in cell death in HK2 cells transfected with mammalian expression plasmids [2].

## Supplementary material and/or additional information

Trypan blue is a widely-available low-cost dye used to distinguish between intact live cells and permeable dead cells and debris [3]. Although this dye is commonly used to stain and exclude dead cells for the quantification of live cells using a hemocytometer, to the best of our knowledge the quantification of cell debris found in the medium using trypan blue has not yet been described. The use of cell debris, a component that is generally discarded in most experiments, to generate data on cell viability status represents the first advantage of this method. Because debris is isolated from the sample, all of the media and cell monolayer remains available for additional experiments.

Other common cell death assays include (a) intracellular protein release assays, such as the LDH release assay, and (b) metabolism-based assays [4,5]. The former relies upon the release of proteins that are normally expressed in cells into the medium as a result of cytotoxicity-induced perturbations in membrane integrity. Activity is then measured in the medium by providing the enzyme with detectable substrate. Because ATP production is reduced in dying cells, metabolism-based assays can also be used to quantify ATP levels in cells using a firefly-luciferase system [6]. Each of these enzyme-dependent classes of cell death assays, however, share a common limitation. The expression of such enzymes can be affected by certain treatments thus leading to either over- or under-estimations of true cell death. Because the TBCD assay relies on damaged cell membrane, which represents a more direct marker of cell viability, this assay is not affected by alterations in enzyme expression and/or activity.

Another common method to quantify cell death in a manner dependent on cell membrane integrity involves a fluorescent dye, known as propidium iodide (PI) [7]. Similar to trypan blue, PI does not readily pass through intact membrane in live cells. In damaged cells, PI passes through pores of the cell membrane and intercalates with DNA; therefore, PI is generally used to visualize and quantify cell death using fluorescence microscopy and/or flow cytometry [8]. High-throughput PI-based fluorometric assays have also been developed, however, these are often expensive and laborious processes that are known to produce false-positive results. Furthermore, as PI-based assay rely on intact nuclei of damaged cells within the cell monolayer, PI assays also fail to detect dead floating cells that have lifted from the monolayer [9]. In this regard, the because the LDH and TBCD assays quantify a by-product of cell death found in the medium, these assays may represent better tools to assess total cell death.

**Fig. 4:**
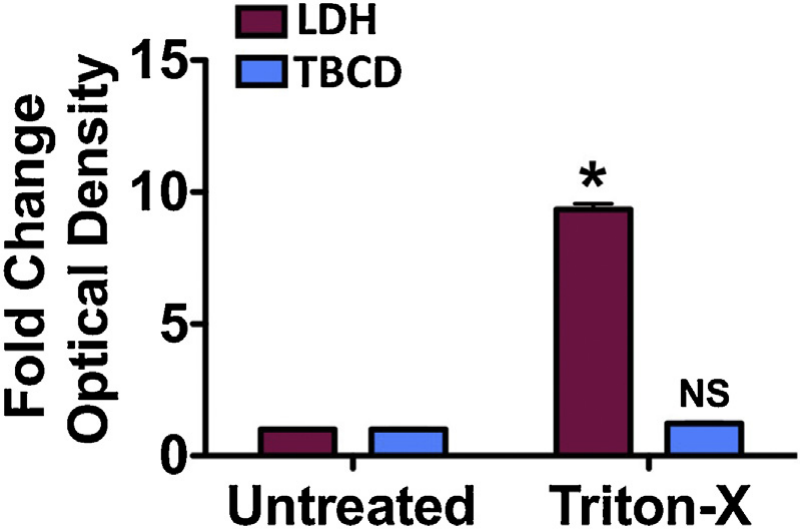
To determine the limitations of the trypan blue cellular debris (TBCD) assay in comparison to the lactate dehydrogenase (LDH) release assay, HuH7 immortalized human hepatocytes were treated with a detergent capable of dissolving cell membrane. Representative images of cells treated with Triton-X were taken with a light microscope. *, p < 0.05 vs. LDH assay - untreated; NS, non-significant vs. TBCD assay untreated. Statistical analysis was carried out using the unpaired Student’s *t*-test. Error bars are presented as SD.

The dependence on cell membrane, however, also has its limitations. Detergent-based treatments known to dissolve cell membrane significantly reduce the ability of the TBCD assay to detect changes in media debris content. To test this, a side-by-side comparison was made between the TBCD assay and the LDH release assay using HuH7 cells treated with Triton-X; a harsh detergent commonly used to generate positive control values for the LDH release assay (Fig. 4). Although nearly 100% cell death occurred as a result of this treatment, which was confirmed using a light microscope, media debris content did not accurately reflect cell death.

In conclusion, there exists many assays for the assessment of cytotoxicity and cell death and each has its limitations. The assay described in this report adds to the repertoire of techniques available to examine cell death and may offer significant advantage over enzyme-based assays in certain circumstances.
